# Post-pandemic anxiety among nurses and midwives: the role of intolerance of uncertainty, chronic conditions, and professional experience

**DOI:** 10.1186/s12912-026-04939-8

**Published:** 2026-06-23

**Authors:** Anila Cake, Ergys Ramosaçaj, Artan Simaku, Agron Bytyqi, Flora Zyberaj

**Affiliations:** 1 Faculty of Technical Medical Sciences – FSHMT, University of Medicine, Tirana, Albania; 2 Institute of Public Health, Tirana, Albania; 3 Patients’ Rights Association in Kosovo, PRAK, Kosovo, Kosovo

**Keywords:** Post-pandemic, Nurses, Midwives, Trait anxiety, Intolerance of uncertainty, Cronic conditions

## Abstract

**Background:**

Although healthcare systems have largely stabilized following the COVID-19 pandemic, the long-term psychological adaptation of nurses and midwives remains insufficiently understood. Persistent exposure to uncertainty and individual health-related vulnerabilities may contribute to sustained anxiety beyond the acute crisis phase.

**Aim:**

This study aimed to assess levels of state anxiety, trait anxiety, and intolerance of uncertainty among nurses and midwives in the post-pandemic period, and to examine whether intolerance of uncertainty, chronic health conditions, and professional experience are associated with elevated trait anxiety.

**Methods:**

A cross-sectional analytical study was conducted among 1,201 nurses and midwives working in public hospitals in Albania. Participants were selected through stratified random sampling, and data were collected from March 2024 to May 2025 using a structured questionnaire adapted into Albanian, including the State–Trait Anxiety Inventory (STAI) and the 12-item Intolerance of Uncertainty Scale (IUS-12). Descriptive and inferential analyses, including group comparisons, correlation analyses, and multiple linear regression, were performed using SPSS. Statistical significance was set at *p* < 0.05.

**Results:**

Participants reported moderate levels of state anxiety (M = 2.15, SD = 0.90), trait anxiety (M = 2.40, SD = 0.86), and intolerance of uncertainty (M = 2.64, SD = 1.01). Trait anxiety was positively correlated with intolerance of uncertainty (*r* = 0.46, *p* < 0.001) and state anxiety (*r* = 0.52, *p* < 0.001). Participants reporting chronic health conditions had higher trait anxiety than those without chronic conditions (M = 2.57 vs. 2.35, *p* < 0.001). In multiple linear regression, intolerance of uncertainty, chronic health conditions, and lower professional experience remained independently associated with trait anxiety; the model explained 26.5% of the variance in trait anxiety (adjusted R² = 0.262, *p* < 0.001).

**Conclusion:**

Nurses and midwives continue to experience moderate levels of anxiety and intolerance of uncertainty in the post-pandemic period, suggesting that psychological adaptation may involve stabilization rather than full recovery. The findings highlight the role of both individual vulnerability and professional context in shaping long-term emotional outcomes and underscore the need for sustained, targeted interventions to support healthcare workforce wellbeing.

## Introduction

The COVID-19 pandemic imposed unprecedented psychological demands on healthcare workers worldwide, particularly nurses and midwives who were continuously exposed to high workloads, uncertainty, and rapidly changing clinical environments [1, 2]. While healthcare systems have transitioned into a more stable post-pandemic phase, the longer-term psychological adaptation of frontline staff remains insufficiently understood [3].

Emerging evidence suggests that the psychological impact of large-scale health crises extends beyond immediate stress reactions, contributing to more persistent patterns of emotional vulnerability [4]. In this context, anxiety is increasingly conceptualized not only as a situational response but also as a more stable psychological tendency shaped by prolonged exposure to uncertainty and individual-level factors [5].

A key construct in this regard is intolerance of uncertainty, defined as a dispositional tendency to perceive uncertain situations as stressful or threatening. This construct has been consistently associated with elevated anxiety across clinical and non-clinical populations [6, 7]. In healthcare environments characterized by unpredictability, evolving protocols, and systemic pressures, intolerance of uncertainty may remain a critical factor influencing mental health even after the acute crisis phase [8, 9].

In addition, individual health status may play an important role in psychological adaptation. Nurses and midwives with chronic conditions may perceive increased personal risk, contributing to sustained anxiety responses [10, 11]. Furthermore, professional experience may influence coping capacity, with less experienced staff potentially more vulnerable to prolonged stress [12].

Despite extensive research on the immediate psychological impact of COVID-19, fewer studies have examined how these factors interact in the post-pandemic context [3, 9]. Understanding these relationships is essential for informing long-term mental health strategies in healthcare settings.

Therefore, this study aimed to assess levels of state anxiety, trait anxiety, and intolerance of uncertainty among nurses and midwives in the post-pandemic period, and to examine whether intolerance of uncertainty, chronic health conditions, and professional experience predict elevated trait anxiety.

## Methods

### Study design and setting

This was a quantitative cross-sectional analytical study conducted among nurses and midwives employed in public hospitals in Albania. The study was carried out during the post-pandemic period, after the acute phases of the COVID-19 pandemic, with the objective of assessing anxiety-related outcomes and intolerance of uncertainty among nurses and midwives. Data collection was conducted from March 2024 to May 2025. The term “post-pandemic period” was used operationally to refer to the phase following the end of the COVID-19 public health emergency of international concern. On 5 May 2023, the World Health Organization declared that COVID-19 no longer constituted a public health emergency of international concern, while emphasizing that COVID-19 remained an ongoing health issue. Therefore, the present data collection period, from March 2024 to May 2025, was considered post-pandemic in relation to the acute emergency phase rather than indicating the complete disappearance of COVID-19 or its consequences [13].

### Participants and sampling

Eligible participants were registered nurses and midwives actively employed in clinical practice in public hospitals in Albania. Participants were required to have at least six months of professional experience in their current role, in order to ensure sufficient exposure to the hospital-based healthcare environment during or after the COVID-19 pandemic. Individuals on long-term medical leave, maternity leave, or not directly involved in patient care during the pandemic period were excluded.

According to national workforce statistics, the eligible professional population included 26,490 nurses, 2,413 midwives, and 2,100 professionals qualified as both nurses and midwives, for a cumulative total of 31,003 nurses and midwives. Based on this population, the required sample size was calculated using a 95% confidence level, an expected proportion of 50% (*p* = 0.5), and a 3% margin of error. The sample size was further adjusted for an anticipated non-response rate of 20%, in order to preserve adequate statistical representation.

A stratified random sampling approach was applied to improve representation of nurses and midwives working in public hospitals. The sampling frame consisted of institutional staff lists or official ward-level rosters of eligible nurses and midwives provided by participating hospitals. Seventeen public hospitals were included, comprising university, regional, and municipal hospitals across Albania. Strata were defined according to professional role (nurse or midwife) and hospital type. Where feasible, proportional allocation was used so that the number of invited participants from each stratum reflected the approximate distribution of eligible nurses and midwives within the participating hospitals. Within each stratum, eligible participants were selected randomly from staff lists or ward rosters. Replacement was permitted only when a selected individual was ineligible, unavailable during the data collection period, or declined participation. This procedure was intended to reduce selection bias and to ensure that the final sample reflected both professional and institutional variation within the public hospital system. Data collection was conducted across 17 university, regional, and municipal hospitals in Albania. A total of 1,201 questionnaires fulfilled predefined quality criteria and were included in the final analysis, corresponding to approximately 3.9% of the national workforce in these professional categories.

Based on the final analytical sample and the planned 20% adjustment for non-response, approximately 1,500 nurses and midwives were estimated to have been approached during the recruitment process. Therefore, the estimated participation proportion was approximately 80.0%. Because the number of initially invited participants was reconstructed from the planned sampling target and the non-response adjustment, the participation proportion should be interpreted as an estimated rather than directly observed response rate. A centralized log of all invited individuals, refusals, and non-returns was not maintained uniformly across hospitals. Consequently, the reported figure provides an approximate indication of recruitment efficiency but should not be interpreted as a formally measured response rate.

### Measures

Sociodemographic and professional characteristics. Participants provided information on age, gender, marital status, years of professional experience, professional role, type of hospital setting, and the presence of self-reported chronic health conditions. These variables were examined descriptively and, where appropriate, as potential predictors or control variables in inferential analyses.

Anxiety. Anxiety was assessed using the State–Trait Anxiety Inventory (STAI), which consists of two 20-item subscales: the State Anxiety subscale (STAI-S) and the Trait Anxiety subscale (STAI-T) [14]. STAI-S measures transient anxiety-related emotional states at the time of assessment, whereas STAI-T assesses a more stable tendency toward anxiety. Items were rated on a 4-point Likert-type scale. Positively worded items were reverse coded according to the scoring instructions so that higher scores consistently reflected higher anxiety. The original distinction between STAI-S and STAI-T was preserved in the analysis.

Intolerance of uncertainty. Intolerance of uncertainty was measured using the 12-item Intolerance of Uncertainty Scale (IUS-12), a standardized instrument that captures cognitive and behavioral responses to uncertain situations. Higher scores indicate greater intolerance of uncertainty.

### Instrument adaptation procedure

The questionnaire was adapted from a previously published study conducted among nurses and midwives during the COVID-19 outbreak in Turkey by Aksoy and Koçak (2020), with permission obtained from the original author [15]. The instrument included the STAI and IUS-12, both of which are internationally used and validated measures.

The questionnaire was translated into Albanian and reviewed for linguistic clarity, conceptual equivalence, and cultural appropriateness by members of the research team with expertise in nursing, public health, epidemiology, and statistics. Particular attention was given to preserving the original meaning of each item while ensuring that wording was understandable in the Albanian healthcare context. Although a full forward–backward translation validation study was not conducted, the translated version underwent expert review and pilot testing before final implementation.

The Albanian version was pilot tested among 20 nurses and midwives who were not included in the final analytical sample. Feedback from the pilot phase was used to refine item wording, questionnaire structure, and formatting. Following the pilot phase, the final questionnaire was prepared in both printed and electronic formats.

### Reliability and validity considerations

Internal consistency was assessed using Cronbach’s alpha coefficient. In the present sample, Cronbach’s alpha was 0.72 for STAI-S, 0.71 for STAI-T, 0.85 for IUS-12, and 0.86 for the total instrument, indicating acceptable to good internal consistency. Content and face validity were supported through expert review by members of the research team with expertise in nursing, public health, epidemiology, and statistics, followed by pilot testing among 20 nurses and midwives who were not included in the final analysis. Construct validity was considered indirectly through the expected positive correlations between trait anxiety, state anxiety, and intolerance of uncertainty. Known-groups validity was explored by comparing trait anxiety according to chronic health condition status and professional experience, based on the theoretical expectation that participants with chronic conditions and less professional experience would report higher anxiety. However, a full psychometric validation of the Albanian version, including confirmatory factor analysis and formal measurement invariance testing, was not performed. Therefore, the validity evidence should be interpreted as preliminary and sample-specific.

### Data collection procedure

The questionnaire was distributed in printed format. Participants completed the self-administered questionnaire independently. Completed questionnaires were collected by head nurses of the respective wards and were subsequently checked for completeness and quality. Head nurses facilitated only the logistical distribution and collection of questionnaires and were not involved in checking individual responses.

### Statistical analysis

Data were analyzed using IBM SPSS Statistics. Descriptive statistics were calculated to summarize sociodemographic and professional characteristics, as well as levels of state anxiety, trait anxiety, and intolerance of uncertainty. Continuous variables were presented as means and standard deviations, while categorical variables were summarized as frequencies and percentages.

Group comparisons were performed using independent samples t-tests or one-way analysis of variance (ANOVA), as appropriate. Pearson correlation analysis was used to examine associations between continuous psychological constructs. Multiple linear regression analysis was conducted to identify predictors of trait anxiety. Trait anxiety was entered as the dependent variable, while intolerance of uncertainty, presence of chronic health conditions, and professional experience were entered as independent variables. Age and gender were included as control variables to account for potential confounding. Statistical significance was set at *p* < 0.05.

Completed questionnaires were reviewed for completeness and internal consistency before data entry. Questionnaires with substantial missing information or inconsistent responses were excluded from the final dataset. For multivariable regression analysis, a complete-case approach was applied; therefore, only participants with complete data for STAI-T and the selected predictor variables were included in the model (*N* = 1,169).

For item-level descriptive analyses, percentages were calculated using the number of valid responses available for each item. For scale-level analyses, composite scores were calculated for participants with sufficient valid responses according to the predefined scoring procedure.

## Results

### Participant characteristics

A total of 1,201 nurses and midwives participated in the study, with a mean age of 39.5 ± 10.9 years. The majority of participants were female (85.2%) and worked primarily in urban hospital settings (89.8%). Most respondents were nurses (80%), while midwives accounted for 19.8% of the sample. In terms of professional experience, 37.9% had between 6 and 15 years of work experience, and 26% had less than five years of experience. Additionally, 22.7% of participants reported the presence of at least one chronic health condition, including hypertension, thyroid disorders, or diabetes.

State anxiety. Participants reported a combination of positive affect and worry-related indicators. Higher endorsements for positive mood and happiness were observed, with 63.9% reporting being in a good mood and 61.4% feeling happy at the time of assessment. Concurrently, 44.3% reported feeling worried and 42.7% expressed concern about future outcomes. Detailed item responses are presented in Table 1, while key state anxiety indicators are summarized in Table 2.

**Table 1:** Distribution of responses to state anxiety items

Item	Not at all *n* (%)	Somewhat *n* (%)	Moderately *n* (%)	Very much *n* (%)
I currently feel calm	363 (30.8)	478 (40.5)	195 (16.5)	143 (12.1)
I feel secure	303 (26.0)	508 (43.6)	238 (20.4)	117 (10.0)
I feel nervous	202 (17.3)	533 (45.6)	363 (31.1)	70 (6.0)
I feel self-pity	478 (40.8)	383 (32.7)	236 (20.1)	76 (6.5)
I feel at ease	389 (33.7)	371 (32.1)	232 (20.1)	163 (14.1)
I am not enjoying things	437 (37.8)	350 (30.3)	178 (15.4)	192 (16.6)
I worry about what will happen to me	218 (18.7)	451 (38.7)	318 (27.3)	179 (15.4)
I feel rested	512 (43.8)	393 (33.6)	196 (16.8)	68 (5.8)
I feel worried	202 (17.3)	447 (38.4)	361 (31.0)	155 (13.3)
I feel comfortable	548 (47.2)	354 (30.5)	178 (15.3)	81 (7.0)
I feel confident	131 (11.4)	378 (32.8)	376 (32.6)	268 (23.2)
I am in a bad mood	246 (21.3)	533 (46.1)	279 (24.2)	97 (8.4)
I feel angry	358 (31.2)	477 (41.6)	206 (17.9)	107 (9.3)
I feel tense	310 (27.0)	469 (40.9)	278 (24.2)	90 (7.8)
I feel relieved	499 (44.6)	348 (31.1)	179 (16.0)	92 (8.2)
I am satisfied with my situation during COVID-19	681 (59.0)	247 (21.4)	143 (12.4)	84 (7.3)
I feel worried now	496 (42.5)	466 (39.9)	148 (12.7)	58 (5.0)
I feel emotionally overwhelmed	488 (42.7)	403 (35.3)	183 (16.0)	69 (6.0)
I feel happy now	162 (13.9)	287 (24.7)	430 (37.0)	284 (24.4)
I am in a good mood now	117 (9.9)	310 (26.2)	404 (34.2)	351 (29.7)

**Table 2:** Key state anxiety indicators (Top items by “moderately/very much”)

State anxiety item	Moderately *n* (%)	Very much *n* (%)	Moderately/Very much *n* (%)
I feel confident	376 (32.6)	268 (23.2)	644 (55.8)
I am in a good mood now	404 (34.2)	351 (29.7)	755 (63.9)
I feel happy now	430 (37.0)	284 (24.4)	714 (61.4)
I worry about what will happen to me	318 (27.3)	179 (15.4)	497 (42.7)
I feel worried	361 (31.0)	155 (13.3)	516 (44.3)
I feel nervous	363 (31.1)	70 (6.0)	433 (37.1)
I feel tense	278 (24.2)	90 (7.8)	368 (32.0)
I am not enjoying things	178 (15.4)	192 (16.6)	370 (32.0)

Trait anxiety. A notable proportion of participants reported recurrent worry and emotional sensitivity. Among selected negative trait anxiety indicators, the highest endorsement was observed for taking everything seriously and worrying, reported often or almost always by 482 participants (41.9%), followed by fatigue in 441 participants (38.5%), worrying about minor matters in 349 participants (29.8%), taking disappointments seriously in 349 participants (30.0%), and recent thoughts making participants nervous in 341 participants (29.2%). Full item distributions are provided in Table 3, while selected negative trait anxiety indicators are summarized in Table 4.

**Table 3:** Distribution of responses to trait anxiety items

Item	Almost never *n* (%)	Sometimes *n* (%)	Often *n* (%)	Almost always *n* (%)
I am usually in a good mood	63 (5.3)	337 (28.4)	556 (46.8)	232 (19.5)
I usually get tired easily	137 (11.6)	669 (56.8)	295 (25.0)	77 (6.5)
I cry easily	269 (23.0)	562 (48.1)	250 (21.4)	87 (7.4)
I want to be as happy as others	55 (4.8)	238 (21.0)	357 (31.4)	484 (42.6)
I miss opportunities because I cannot decide quickly	236 (20.3)	604 (52.1)	234 (20.2)	86 (7.4)
I feel fatigued	164 (14.3)	543 (47.3)	352 (30.7)	89 (7.8)
I am usually calm and composed	72 (6.1)	367 (31.3)	464 (39.6)	268 (22.9)
Difficulties accumulate beyond my control	265 (22.7)	623 (53.5)	229 (19.7)	48 (4.1)
I worry about minor matters	293 (25.0)	530 (45.2)	286 (24.4)	63 (5.4)
I am usually happy	55 (4.8)	323 (28.5)	510 (44.9)	247 (21.8)
I take everything seriously and worry	154 (13.4)	514 (44.7)	335 (29.1)	147 (12.8)
I usually feel insecure	325 (27.8)	575 (49.1)	196 (16.8)	74 (6.3)
I usually feel secure	99 (8.5)	367 (31.4)	508 (43.5)	194 (16.6)
I avoid confronting difficult situations	264 (22.6)	440 (37.7)	316 (27.1)	144 (12.3)
I usually feel sad	335 (28.8)	582 (50.0)	182 (15.6)	65 (5.6)
I am generally satisfied with my life	78 (6.7)	307 (26.5)	462 (39.9)	312 (26.9)
Minor thoughts bother me	341 (29.5)	476 (41.2)	232 (20.1)	105 (9.1)
I take disappointments seriously	380 (32.6)	438 (37.5)	227 (19.5)	122 (10.5)
I am a determined person	98 (8.4)	228 (19.5)	437 (37.4)	407 (34.8)
Things that have recently crossed my mind make me nervous	340 (29.1)	488 (41.7)	234 (20.0)	107 (9.2)

**Table 4:** Selected negative trait anxiety indicators by “often/almost always” responses

Trait anxiety item	Often *n* (%)	Almost always *n* (%)	Often/Almost always *n* (%)
I take everything seriously and worry	335 (29.1)	147 (12.8)	482 (41.9)
I feel fatigued	352 (30.7)	89 (7.8)	441 (38.5)
I worry about minor matters	286 (24.4)	63 (5.4)	349 (29.8)
I take disappointments seriously	227 (19.5)	122 (10.5)	349 (30.0)
Things that have recently crossed my mind make me nervous	234 (20.0)	107 (9.2)	341 (29.2)
I cry easily	250 (21.4)	87 (7.4)	337 (28.8)
I miss opportunities because I cannot decide quickly	234 (20.2)	86 (7.4)	320 (27.6)

### Intolerance of uncertainty

Participants reported moderate levels of intolerance of uncertainty. The highest mean score was observed for the item reflecting the need to organize situations in detail in advance (M = 3.22, SD = 1.25), followed by the need to anticipate future events (M = 2.91, SD = 1.33).

Lower mean scores were observed for items reflecting direct functional impairment, such as difficulty acting under uncertainty (M = 2.26, SD = 1.24) and perceived restriction in daily functioning (M = 2.36, SD = 1.27).

Standard deviations ranging from 1.20 to 1.35 indicated variability in responses across participants.

Mean responses for the Intolerance of Uncertainty Scale (IUS-12) are presented in Table 5.

**Table 5:** Mean scores for intolerance of uncertainty items

Item	Mean (M)	Standard Deviation (SD)
Unexpected events upset me greatly	2.59	1.20
I get frustrated if I do not have all the information I need	2.75	1.20
I always need to look ahead to avoid surprises	2.98	1.23
Even small unexpected events can disrupt everything	2.50	1.22
I want to know what the future holds for me	2.91	1.33
I cannot tolerate being caught off guard	2.66	1.28
I need to organize everything in detail in advance	3.22	1.25
Uncertainty prevents me from living life fully	2.36	1.27
Uncertainty makes it difficult to act	2.26	1.24
I cannot function well when things are uncertain	2.58	1.29
Even small doubts interfere with my actions	2.26	1.31
I avoid uncertain situations	2.64	1.35

**Fig. 1:**
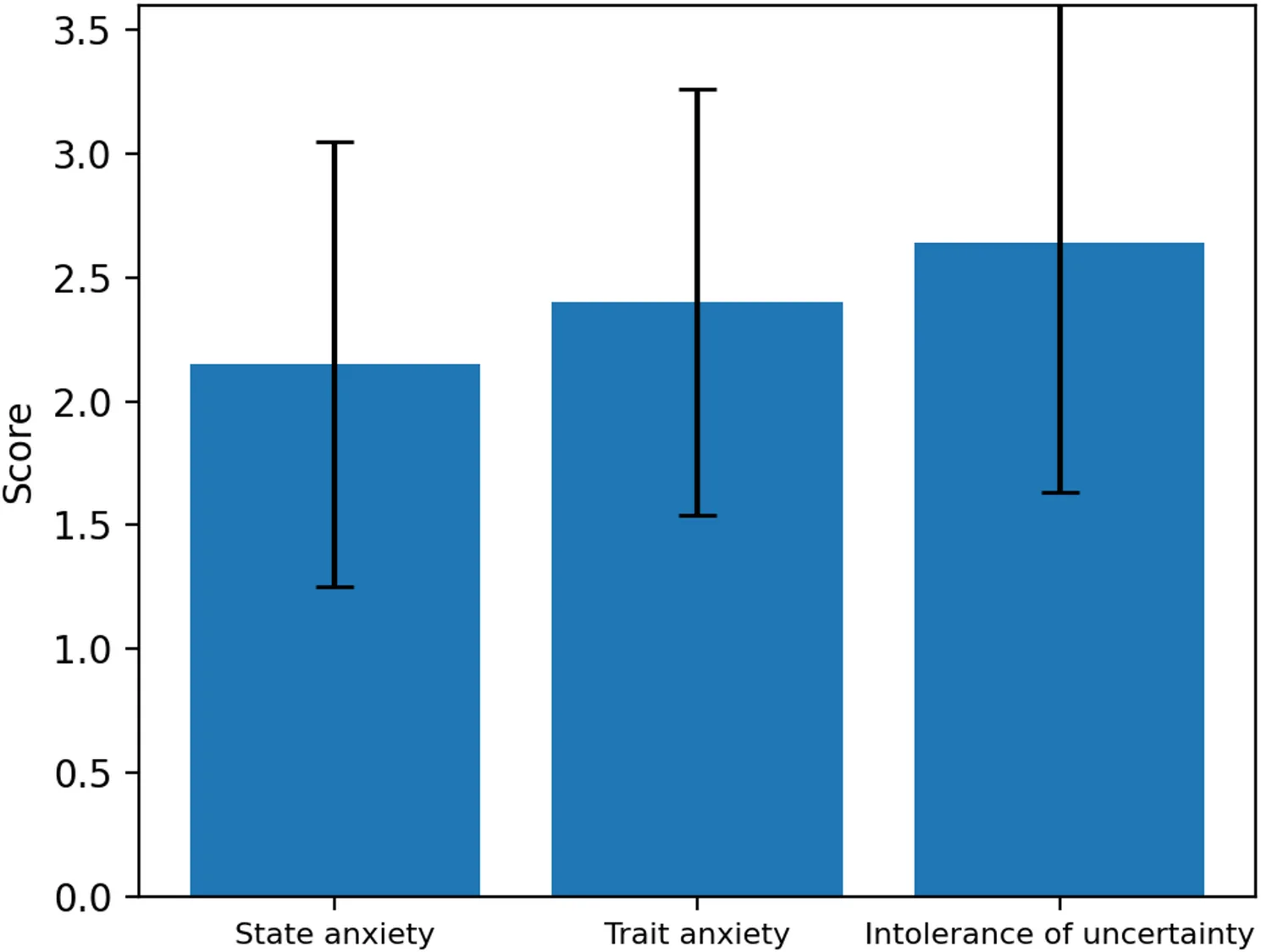
Mean scores and standard deviations for state anxiety, trait anxiety, and intolerance of uncertainty

Overall pattern. State anxiety demonstrated a moderate level (M = 2.15, SD = 0.90), while trait anxiety showed a slightly higher mean score (M = 2.40, SD = 0.86). Intolerance of uncertainty also remained within the moderate range (M = 2.64, SD = 1.01). Mean scores across constructs are summarized in Table 6 and illustrated in Fig. 1.

**Table 6:** Summary of mean scores across study constructs

Construct	Mean (M)	Standard Deviation (SD)
State anxiety	2.15	0.90
Trait anxiety	2.40	0.86
Intolerance of uncertainty (IUS-12)	2.64	1.01

### Inferential analyses

Additional analyses were performed to examine group differences, bivariate associations, and independent predictors of trait anxiety.

### Group comparisons

Participants with self-reported chronic health conditions had higher trait anxiety scores than participants without chronic conditions (M = 2.57, SD = 0.82 vs. M = 2.35, SD = 0.86; t = 3.84, *p* < 0.001). Trait anxiety also differed by professional experience group, with the highest mean score observed among participants with less than five years of experience (F = 11.62, *p* < 0.001). Female participants showed slightly higher mean trait anxiety than male participants (M = 2.42 vs. 2.30; *p* = 0.037). The corresponding group comparisons are presented in Table 7.

**Table 7:** Group comparisons for trait anxiety

Variable / group	*n*	Trait anxiety, M (SD)	Test statistic	*p*-value
Chronic condition: Yes	273	2.57 (0.82)	t = 3.84	< 0.001
Chronic condition: No	928	2.35 (0.86)		
Professional experience < 5 years	312	2.55 (0.84)	F = 11.62	< 0.001
Professional experience 6–15 years	455	2.42 (0.85)		
Professional experience > 15 years	434	2.29 (0.86)		
Female	1,023	2.42 (0.86)	t = 2.09	0.037
Male	178	2.30 (0.83)		

### Correlation analyses

Trait anxiety was moderately and positively correlated with intolerance of uncertainty (*r* = 0.46, *p* < 0.001) and state anxiety (*r* = 0.52, *p* < 0.001). Professional experience showed a weak negative correlation with trait anxiety (*r* = -0.18, *p* < 0.001), indicating lower trait anxiety among more experienced participants. Age was weakly and inversely correlated with trait anxiety (*r* = -0.09, *p* = 0.014). The correlation matrix for the main continuous variables is presented in Table 8.

**Table 8:** Pearson correlation matrix for main continuous variables

Variable	1	2	3	4	5
1. Trait anxiety	1.00				
2. State anxiety	0.52***	1.00			
3. Intolerance of uncertainty	0.46***	0.39***	1.00		
4. Professional experience, years	-0.18***	-0.11**	-0.14***	1.00	
5. Age, years	-0.09*	-0.06	-0.08*	0.63***	1.00

Note. *p < 0.05; **p < 0.01; ***p < 0.001

Multiple linear regression. Complete data for STAI-T and all predictors were available for 1,169 participants. The regression model was statistically significant (F[5,1163] = 83.86, *p* < 0.001) and explained 26.5% of the variance in trait anxiety (R² = 0.265; adjusted R² = 0.262). Intolerance of uncertainty was the strongest independent predictor of higher trait anxiety (β = 0.45, *p* < 0.001). The presence of chronic health conditions was also independently associated with higher trait anxiety (β = 0.12, *p* < 0.001), whereas greater professional experience was associated with lower trait anxiety (β = -0.10, *p* = 0.004). Age and gender were not statistically significant after adjustment for the other predictors. The full regression model is presented in Table 9.

**Table 9:** Multiple linear regression model predicting trait anxiety

Predictor	B	SE	β	t	*p*-value
Intolerance of uncertainty	0.38	0.03	0.45	12.96	< 0.001
Chronic health condition (yes vs. no)	0.23	0.06	0.12	3.94	< 0.001
Professional experience, years	-0.009	0.003	-0.10	-2.91	0.004
Age, years	-0.003	0.003	-0.04	-1.19	0.236
Gender (female vs. male)	0.11	0.07	0.05	1.61	0.108

Model summary: *N* = 1,169; F(5,1163) = 83.86; *p* < 0.001; R² = 0.265; adjusted R² = 0.262

Taken together, these inferential findings support the analytical framing of the study by showing that trait anxiety was not only descriptively elevated but also statistically associated with intolerance of uncertainty, chronic health conditions, and professional experience.

## Discussion

This study examined levels of state anxiety, trait anxiety, and intolerance of uncertainty among nurses and midwives in the post-pandemic period. The findings revealed moderate levels across all three constructs, suggesting that psychological burden persists even after the stabilization of healthcare systems. These findings are consistent with previous research examining long-term mental health outcomes among healthcare workers following major health crises [16]. These findings indicate that the impact of the COVID-19 pandemic on nurses and midwives extends beyond the acute crisis phase and may continue to influence emotional wellbeing. At the item level, selected negative trait anxiety indicators suggested that recurrent worry, fatigue, emotional sensitivity, and difficulty managing recent concerns remained relevant components of the trait anxiety profile.

A key finding of this study is the distinction between state anxiety and trait anxiety. While state anxiety remained at moderate levels, trait anxiety was slightly higher, reflecting a more stable and enduring tendency toward emotional distress. This pattern supports existing literature suggesting that prolonged exposure to large-scale health crises may lead to longer-term changes in emotional regulation and psychological functioning [4, 8]. Rather than representing a temporary response, anxiety in this context appears to reflect a more persistent dispositional pattern.

The observed moderate levels of intolerance of uncertainty further reinforce this interpretation. Participants demonstrated an increased need for predictability, structure, and anticipatory control, without exhibiting severe functional impairment. Previous research has consistently identified intolerance of uncertainty as a central factor associated with anxiety across both clinical and occupational contexts [5, 7]. In healthcare environments characterized by ongoing uncertainty, evolving protocols, and systemic pressures, this tendency may contribute to the maintenance of emotional distress even after the acute phase of the pandemic has passed [8]. Recent studies have further confirmed the role of intolerance of uncertainty and resilience mechanisms in shaping post-pandemic mental health outcomes among nurses and midwives [15, 17].

Importantly, the study identified significant associations between trait anxiety, chronic health conditions, and professional experience. The inferential analyses further supported the descriptive findings by showing that trait anxiety was not randomly distributed across the study population, but was associated with specific psychological and professional factors. In the bivariate analyses, trait anxiety showed a moderate positive correlation with intolerance of uncertainty, indicating that participants who reported greater difficulty tolerating unpredictable situations also tended to report higher levels of dispositional anxiety. This finding is theoretically consistent with models of anxiety in which intolerance of uncertainty acts as a cognitive vulnerability factor that amplifies worry, anticipatory distress, and perceived lack of control.

Group comparison analyses also suggested that anxiety levels differed according to individual vulnerability and professional background. Participants reporting chronic health conditions had higher mean trait anxiety scores than those without chronic conditions, supporting the interpretation that perceived personal health vulnerability may continue to influence psychological wellbeing even after the acute pandemic period. This interpretation is consistent with previous evidence showing that perceived health risk and vulnerability were associated with fear and anxiety responses during the COVID-19 crisis [18]. Similarly, less experienced professionals appeared to report higher anxiety levels compared with those with longer professional experience, suggesting that accumulated clinical exposure and professional maturity may contribute to greater emotional regulation and coping capacity.

The multiple linear regression model provided additional evidence that intolerance of uncertainty remained an independent predictor of trait anxiety after adjustment for sociodemographic variables. This suggests that uncertainty-related cognitive vulnerability may have a specific contribution to post-pandemic anxiety beyond general demographic characteristics. Chronic health conditions and lower professional experience also contributed to the model, although their effects were weaker than that of intolerance of uncertainty. These findings indicate that post-pandemic anxiety among nurses and midwives is likely shaped by the combined influence of psychological disposition, perceived health vulnerability, and professional experience.

From an applied perspective, these results suggest that institutional mental health interventions should not focus only on general stress reduction, but should also target uncertainty management, coping strategies, social support, and resilience-building, which have been emphasized as important protective factors for healthcare staff during and after the COVID-19 pandemic [11]. Early-career nurses and midwives, as well as professionals with chronic health conditions, may benefit from more structured psychological support, mentoring, and occupational health monitoring. Such interventions may be particularly relevant in healthcare systems that continue to face organizational instability, workforce shortages, and evolving clinical demands after the COVID-19 pandemic.

When compared with studies conducted during the acute phases of the COVID-19 pandemic, which reported elevated levels of state anxiety and acute psychological distress [1, 2], the present findings suggest a shift toward more stable and enduring patterns of anxiety in the post-pandemic context. This transition indicates that psychological responses may evolve over time, moving from acute stress reactions to more persistent emotional tendencies influenced by both individual and contextual factors. Systematic and rapid reviews continue to highlight the persistent psychological burden among healthcare workers beyond the acute phase of the pandemic, including anxiety, depression, distress, and occupational strain [19–21].

From a professional and organizational perspective, these findings highlight the importance of conceptualizing psychological adaptation as a long-term process rather than a simple return to pre-crisis baseline functioning. Even in the absence of emergency conditions, underlying emotional vulnerability may persist and continue to affect performance, decision-making, and overall wellbeing among healthcare staff.

Overall, this study contributes to the existing literature by focusing on the post-pandemic period and by examining the combined role of intolerance of uncertainty and individual vulnerability factors in shaping sustained anxiety among nurses and midwives [22]. It provides further evidence that the psychological consequences of global health crises may extend well beyond their immediate impact.

### Strengths and limitations

This study benefits from a large and diverse sample of nurses and midwives across multiple hospital-based clinical settings, enhancing the robustness and ecological validity of the findings. The inclusion of nurses and midwives from university, regional, and municipal hospitals allows for a broader understanding of psychological responses across different hospital-based clinical environments.

However, several limitations should be acknowledged. The cross-sectional design limits the ability to draw causal inferences regarding the relationships between variables. Although a stratified sampling approach was used to improve representativeness, potential selection bias cannot be fully excluded because participation was voluntary and depended on institutional and individual willingness to participate. In addition, the participation rate was estimated from the planned recruitment target rather than directly measured from a standardized screening log of all invited and non-responding individuals. Therefore, generalizability should be interpreted with caution. Additionally, reliance on self-reported measures may be subject to response bias, including social desirability and recall bias. In addition, because questionnaires were distributed and collected through head nurses, some participants may have felt indirect institutional pressure or may have been less willing to report anxiety-related symptoms openly, despite the anonymous nature of the questionnaire. The multivariable regression analysis was based on complete cases only; complete data for STAI-T and all selected predictors were available for 1,169 participants, and the findings should therefore be interpreted in relation to the complete-case analytical dataset. Finally, cultural and organizational factors specific to the study context may influence perceptions of uncertainty and anxiety, and should be considered when interpreting the findings. A further limitation is that the Albanian version of the instruments was not subjected to a full psychometric validation study; therefore, the reliability estimates reported in this study should be interpreted as internal consistency evidence for this sample rather than complete validation evidence.

### Implications for practice

The findings of this study suggest that psychological support strategies in the post-pandemic period should extend beyond addressing acute stress and instead focus on long-term adaptation processes. Interventions aimed at improving tolerance of uncertainty, strengthening coping mechanisms, and enhancing resilience may be particularly beneficial.

Special attention should be directed toward early-career nurses and midwives and those with chronic health conditions, as these groups appear to be more vulnerable to sustained anxiety. Organizational policies that promote supportive work environments, ongoing mental health monitoring, and targeted interventions may play a critical role in maintaining workforce wellbeing in the post-pandemic era.

## Conclusion

The findings of this study indicate that nurses and midwives in the post-pandemic period continue to experience moderate levels of anxiety and intolerance of uncertainty, suggesting that psychological adaptation following prolonged systemic stress may involve stabilization rather than full recovery.

Importantly, anxiety in this context appears to reflect a more enduring dispositional tendency rather than a transient situational response. The observed associations between intolerance of uncertainty, chronic health conditions, and professional experience highlight the combined role of individual vulnerability and professional context in shaping long-term emotional outcomes. The inferential findings further suggest that intolerance of uncertainty is the strongest psychological factor associated with trait anxiety, while chronic health conditions and lower professional experience represent additional vulnerability factors. These results support the need for targeted post-pandemic mental health strategies that address uncertainty tolerance, resilience, and professional support, particularly among less experienced nurses and midwives and those with underlying health conditions.

These results emphasize that the psychological impact of large-scale health crises may extend beyond the acute phase and become embedded in everyday professional functioning. Future research should adopt longitudinal approaches to better understand the evolution of anxiety over time and to identify mechanisms that support long-term psychological adaptation in healthcare settings.

## Data Availability

The datasets generated and/or analyzed during the current study are not publicly available due to ethical and confidentiality restrictions but are available from the corresponding author on reasonable request.
